# The Role of the Tumor Microenvironment and Treatment Strategies in Colorectal Cancer

**DOI:** 10.3389/fimmu.2021.792691

**Published:** 2021-12-02

**Authors:** Yaping Chen, Xiao Zheng, Changping Wu

**Affiliations:** ^1^ Department of Tumor Biological Treatment, The Third Affiliated Hospital of Soochow University, Changzhou, China; ^2^ Department of Oncology, The Third Affiliated Hospital of Soochow University, Changzhou, China

**Keywords:** immunotherapy, immune microenvironment, bevacizumab, PD-1, colorectal cancer

## Abstract

Colorectal cancer (CRC) has the second highest mortality rate among all cancers worldwide. Surgery, chemotherapy, radiotherapy, molecular targeting and other treatment methods have significantly prolonged the survival of patients with CRC. Recently, the emergence of tumor immunotherapy represented by immune checkpoint inhibitors (ICIs) has brought new immunotherapy options for the treatment of advanced CRC. As the efficacy of ICIs is closely related to the tumor immune microenvironment (TME), it is necessary to clarify the relationship between the immune microenvironment of CRC and the efficacy of immunotherapy to ensure that the appropriate drugs are selected. We herein review the latest research progress in the immune microenvironment and strategies related to immunotherapy for CRC. We hope that this review helps in the selection of appropriate treatment strategies for CRC patients.

## Introduction

Colorectal cancer (CRC) is a common malignant tumor of the digestive system. Worldwide, CRC ranks third in the incidence rate and second in the mortality rate among malignant tumors (1). The incidence of CRC is related to many factors, such as heredity, a low-fiber diet, smoking, lack of exercise, and obesity. At present, changes in the intestinal microbiome and metabolites are also considered risk factors for CRC (2). Traditionally, the treatment of CRC includes surgery, chemotherapy and radiotherapy. In recent years, along with the continuous research on the embryonic origin, anatomical structure, tumor clinical manifestations and genes of the left and right colon, there have been different targeted therapies for “left and right CRC dispute”, namely, cetuximab and bevacizumab, respectively. Furthermore, the advent of the small molecule anti-vascular oral drug, apatinib mesylate, has diversified the CRC treatment. Nevertheless, the prognosis of CRC depends on the stage of the tumor. The mortality of stage I/II is 8-13%, that of stage III is 11-47%, and that of stage IV is as high as 89% (3). Early detection of tumors and early effective treatment can reduce the mortality of CRC. In addition to the small molecule anti-vascular oral drugs, new therapeutic strategies for the treatment of advanced CRC based on immune checkpoint inhibitors (ICIs) have progressed. Overall, advancements in research on the tumor immune microenvironment (TME) and strategies related to immunotherapy are expected to provide more treatment choices for CRC patients.

CRC is a typical tumor infiltrated by effector memory lymphocytes, yet there have been no major breakthroughs in clinical prognosis and immunotherapy (4). It is essential to elucidate the immune environment of CRC to improve current treatment strategies and prognosis for CRC patients. The TME refers to the environment of tumor cells or tumor stem cells, which is closely related to tumor occurrence, development and metastasis of tumors. In the TME, angiogenesis is induced, and regulatory T cells (Tregs) and myeloid-derived suppressor cells (MDSCs) are recruited, which helps suppress the antitumor immune response and promote tumor progression. Furthermore, cytokines within the TME manipulate immune functions and are involved in muted immune responses that guide tumor progression (5). Therefore, the TME of CRC is an important factor affecting cancer immunotherapy. It is necessary to develop a comprehensive understanding of the TME of CRC and extend this knowledge to current treatment strategies that target dysfunctional components in the TME. This paper reviews the interaction of various components of the CRC microenvironment and related treatment strategies, with the goal of finding more effective treatment methods through a better understanding of the interaction of CRC microenvironment.

## The Colorectum Composition and Microenvironment

The colorectum is not only the main digestive organ but also an important immune organ, participating in innate and adaptive immunity. The colorectum includes the intestinal epithelium, intestinal intraepithelial lymphocytes and lamina propria lymphocytes. The intestinal epithelium digests and absorbs nutrients and forms a mucosal barrier, which effectively prevents the invasion of harmful bacteria in the intestine (6, 7). The intestinal epithelium is mainly composed of absorptive columnar epithelial cells, goblet cells and endocrine cells. Goblet cells secrete a variety of mucus proteins to form a mucus barrier to limit the invasion of bacteria into the intestinal mucosa. If the intestinal tract lacks mucus, long-term exposure to the bacterial environment may cause chronic inflammation similar to ulcerative colitis, possibly leading to cancer (8, 9). Intestinal epithelial cells also participate in the immune response. These cells obtain lumen antigens and present them to dendritic cells (DCs) in the intestinal lamina propria, which is called the goblet cell associated antigen channel (gap cells) (10, 11). McDonald et al. found that interfering with the interaction between APCs and intestinal epithelial cells *via* CCR6- deficiency in mice reduces the transfer of goblet cell products to APCs and induces the mucosal response (12). Goblet cells also regulate the immune response by secreting various cytokines, such as IL25, IL18, IL17, IL15, IL13, IL7 and IL6, as well as the chemokine exotoxins CCL6, CCL9 and CCL20 (10). Therefore, goblet cells play a unique and indispensable role in maintaining intestinal immune homeostasis by interacting with immune cells. Many endocrine cells are distributed in the colon and rectum and act as the sensory sentinels of the intestinal environment and coordinators of mucosal immunity (13). Intestinal endocrine cells can secrete cholecystokinin secretin, somatostatin, and histamine, among others, under the stimulation of chemicals or other molecules. Intestinal endocrine cells are the key receptors of metabolites of intestinal flora. Indeed, endocrine cells recognize pathogen-associated molecular patterns (PAMPs) and release cytokines and peptide hormones, which directly affect the function of the intestinal barrier. In the immune system, peptide hormones such as GLP-1 can regulate the activation of intestinal immune cells (14–16). M cells, also called microfold cells or membranous cells, are located between the lymphoid follicular epithelium and scattered among intestinal mucosal epithelial cells. M cells actively transport a variety of substances, such as soluble antigens and microorganisms, *via* liquid pinocytosis and receptor-mediated endocytosis. Recently, it was found that M cells play a specialized antigen transport role in the mucosal immune system, transporting antigens from the intestinal cavity to the subepithelial lymphoid tissue, so as to induce an immune mucosal immune response or immune tolerance (17, 18). M cell-dependent antigen uptake is mediated by specific receptors, such as β1 integrin, cellular prion protein and glycoprotein-2 (GP2) (17).

The immune cells involved in colorectal mainly include intestinal intraepithelial lymphocytes (IELs) and lamina propria lymphocytes which play an immunomodulatory role in maintaining colorectal homeostasis. The former cells express a variety of receptors, such as chemokine receptor CCR9 and integrin αEβ7. CCR9 interacts with CCL25 produced by the intestinal epithelial cells to help recruit IELs to the intestinal mucosa. Integrin αEβ7 (αE, also known as CD103) interacts with E-cadherin on intestinal cells to promote entry and retention in the intestinal epithelium (19). Approximately 90% of IELs are T cell receptor (TCR) positive, although a small number are TCR negative (20).

Lamina propria lymphocytes include DCs, intestinal T cells and plasma cells. DCs are antigen-presenting cells that are not evenly distributed in the intestine. CD11c^+^CD11b^-^CD103^+^ DCs are commonly found in the colon of mice, whereas CD11b^+^CD103^+^ DCs are commonly found in the small intestine (21). Human intestinal DCs display more complex markers than mouse intestinal DCs. Human CD103^+^ signal regulatory protein α (SIRPα)^-^ intestinal DCs are associated with mouse intestinal CD11b^-^CD103^+^ DCs, whereas human CD103^+^ SIRPα^+^ DCs are closely related to mouse intestinal CD103^+^CD11b^+^ DCs, which regulate the induction of T cells (22). In recent years, it has been found that DCs and goblet cells can interact and participate in the immune response. CD11c^+^CD103^+^ DC subsets present antigens from goblet cells (23). Intestinal T cells include γδ T and αβ T cells. On the one hand, intestinal T cells secrete IFN-γ, TNF-α and other cytokines participating in the immune response against infection and resisting intestinal bacterial immersion. On the other hand, they secrete a variety of factors such as IL-4, IL-5, IL-10, IL-17 and IL-22, to maintain intestinal immune balance. There are also special Treg cells in the intestinal lamina propria that produce IL-10 and TGF-β to negatively regulate the activation of effector T cells and suppress the intestinal inflammation. Plasma cells are distributed in the lamina propria of colorectal tissue and produce different antibodies. In the duodenum/jejunum, 79% of plasma cells express IgA, 18% of plasma cells express IgM and 3% of plasma cells express IgG, while the corresponding numbers in the colon are 90%, 6 and 4%, respectively (24).

## TME in CRC

The components of the TME in CRC include tumor cells, blood vessels, the extracellular matrix, fibroblasts, lymphocytes, bone marrow-derived suppressor cells and signaling molecules.

### Extracellular Matrix (ECM)

The ECM is composed of glycoprotein, collagen, elastin, proteoglycan and other macromolecules, which support and connect tissues and maintain normal physiological functions (25). Compared with normal tissue, the ECM structure of tumor tissue is disordered, and the process by which the infiltration of fibroblasts/myofibroblasts and the subsequent accumulation of significant amounts of collagenous ECM is observed in the TME is called desmoplasia (26). Desmoplasia is connected with poor prognosis and resistance to therapy (27). Furthermore, an abnormal ECM regulates the epithelial-mesenchymal transition (EMT) and affects cancer progression by directly promoting cell transformation and metastasis (28). Wei et al. found that increasing the stiffness of the surrounding ECM drives the EMT in breast cancer cells by promoting TWIST1 translocalization into the nucleus (29). ECM abnormalities also affect the efficacy of immunotherapy *via* dense EMC, preventing not only immune cells from reaching the tumor cells but also immunotherapeutic drugs from reaching the tumor. In addition, the shielding diffusion barrier that the ECM forms result in hypoxia, which directly enhances immune escape by upregulating immunomodulatory factors and increasing angiogenic signals (30). In general, ECM abnormalities relieve the behavioral regulation of stromal cells and promote tumor-related angiogenesis and inflammation, resulting in resistance to immunotherapy in the TME (31).

Peptidylarginine deiminase 4 (PAD4) is a member of the PAD family including calcium dependent isozymes (PADs 1-4 and 6) (32). PAD4 overexpression is typically involved in elevated tumor citrullination and hypercitrullination alters cell-matrix adhesion and enhances metastasis (33). Yuzhalin et al. found that citrullination of the ECM and expression of PAD4 promote liver metastasis of human CRC, which may create opportunities for the development of biomarkers and therapeutic targeting (34). Tenascin C (TNC) is a glycoprotein in the extracellular matrix, and plays a role in promoting metastasis, modulating adhesion and motility, developing angiogenesis, and establishing immune tolerance (35). In addition, Murakami et al. reported that the TNC on the CRC interstitial ECM is a factor driving liver metastasis (36), and differences in the expression of ECM-related proteins, such as the upregulated expression of TNC, exist in patients with liver metastasis and CRC recurrence (37).

### Angiogenesis

Angiogenesis refers to the production of new blood vessels, while tumor angiogenesis is an endless vicious cycle that cannot be self-regulated. After tumorigenesis, cells proliferate rapidly, and the tumor becomes ischemic and hypoxic. Ischemic and hypoxic cancer cells secrete vascular endothelial growth factor (VEGF), which binds to vascular endothelial growth factor receptors (VEGFRs) on the adjacent vascular endothelium to directly stimulate tumor angiogenesis and promote the migration of endothelial cells (38). The basement membrane cells degrade, and the surrounding vascular endothelial cells proliferate rapidly and migrate to the tumor tissue *via* angiogenesis. Angiogenesis of tumor tissue is the result of the joint action of cancer cells, various tumor-related cells and their bioactive products, such as cytokines, growth factors and microbubbles. Various immune cells such as macrophages, neutrophils, immature myelocytes, B cells, T cells and peripheral cells interact in tumor angiogenesis (39).

VEGF is the most important growth factor regulating angiogenesis in colon cancer and is expressed in all colon carcinoma surgical specimens, including normal mucosa, primary colon cancers and metastatic tumors, as well as in human colorectal cancer cell lines (40, 41). Colon cancer patients with high VEGF expression had a significantly worse prognosis than those with low VEGF expression (41). Furthermore, VEGF has three receptors on CRC cells. VEGFR-1 is associated with tumor grade, Dukes stage and lymph node involvement, and VEGFR-2 is correlated with lymph node involvement while no correlation with any of the clinicopathological variables was found for VEGFR-3 (42). Witte et al. found that the expression of VEGFR-3 in >25% of colorectal cancer cells was associated with a significantly poorer overall survival, but not with lymph node metastasis or depth of tumor invasion (43). Overall, angiogenesis is an important mechanism for the occurrence and development of CRC. Tumor cells secrete VEGF and promote tumor-related angiogenesis which further promotes proliferation and distant metastasis, seriously affecting the prognosis of tumor patients.

### Cancer-Associated Fibroblasts (CAFs)

Fibroblasts are nonepithelial, nonvascular and nonhematopoietic cells in connective tissue that are mainly responsible for the formation of extracellular stroma. Fibroblasts maintain the epithelial homeostasis of normal tissues and play an important role in wound healing. When mechanical injury occurs or radiation, temperature, toxins and pathogens cause acute injury, body cells stimulate the protective system, macrophages produce transformation and growth factor-β (TGF-β) and platelet-derived growth factor (PDGF), and fibroblasts and immune cells proliferate and promote angiogenesis (44). CAFs are important components of the TME and play an essential role in tumorigenesis and development. CAFs have many potential origins, but most are considered to originate from local ancestors (45). Tumor cells are usually derived from fibroblasts in tissues, which are induced and activated by tumor cells in the microenvironment (46–48). CAFs interact with tumor cells to promote tumor growth and maintain their malignant tendency. Tumor cells affect the recruitment of CAF precursors and induce normal fibroblasts to differentiate into CAFs, which secrete a variety of growth factors such as TGF-β, VEGF, chemokines and cytokines, such as CXCL12 (SDF-1), CXCL14, CXCL16, CCL2, CCL5, IL-4, and IL-6, and metalloproteinases, such as MMP-1, MMP-2, MMP-3, MMP-9, MMP-13 and MMP-14. These factors stimulate tumor growth, angiogenesis, invasion and metastasis through a variety of mechanisms, thus affecting tumor prognosis.

Endoglin, which is expressed in CAFs in CRC specimens, metastatic lymph nodes and liver metastases, is a member of the TGF-β family of co-receptors and is involved in CAFs-mediated invasion and metastasis through TGF-β signaling pathway activation (49). Hu et al. found that CAFs can directly secrete exosomes to enhance the cell stemness and epithelial-mesenchymal transformation in CRC cells to promote CRC metastasis and chemotherapy resistance. The mechanism is dependent on increased expression of miR-92a-3p, which directly inhibits Fbxw7 and moap1 and activates Wnt/β-Catenin pathway to inhibit mitochondrial apoptosis and promote stem cell differentiation, the EMT, metastasis and 5-FU/L-OHP resistance in CRC cells (50). According to Heichler et al., CAFs secrete IL-6/IL-11 by activating STAT3 signaling pathway to promote tumor development. Moreover, the expression of pSTAT in CRC correlates with patient survival (51). CAFs are related to resistance in gastrointestinal tumors. The fibroproliferative response induced by CAFs interferes with the delivery of drugs to gastrointestinal cancer cells and causes drug resistance to chemotherapy (52).

### Tumor-Associated Macrophages (TAMs)

Macrophages are resident phagocytes in lymphoid and nonlymphoid tissues that participate in steady-state tissue homeostasis by scavenging apoptotic cells and growth factors. Macrophages have a wide range of pathogen recognition receptors, which enable them to effectively phagocytize and induce the production of inflammatory cytokines. It is well known that the TME is rich in macrophages, and TAMs are considered the most abundant immune cell population in solid tumor tissues (53). TAMs are mainly recruited from the periphery by chemokines released from tumor tissues, including CCL2, CCL3, CCL4, CCL5 and CXCL12. These factors bind to corresponding receptors for recruitment of monocytes/macrophages (54). TAMs play an important role in promoting tumor angiogenesis and express a variety of growth factors (such as VEGF, PDGF and bFGF), membrane binding molecules and soluble proteases (including MMPs and cathepsin), inflammatory cytokines (TNF-α, IL-1β, IL-6), cyclooxygenase 2 (COX2) and CXC-chemokine ligand 8 (CXCL8) to promote sustained cell activation and proliferation, promoting ECM remodeling and recruitment and activation of angiogenic cells (55–57).

In CRC, TAMs are enriched in the high incidence site of the epithelial mesenchymal transformation (EMT). TAMs promote the growth and invasion of colon cancer cells through EMT remodeling (58). When HT-29 or HCT116 cells are co-cultured with TAMs (THP-1 cells stimulated by conditioned medium from a CRC cell line), TAM derived IL-6 activates the JAK2/STAT3 pathway, which results in increased FoxQ1 expression, leads to the production of CCL2 and promotes the recruitment of macrophages, thus enhancing the migration and invasion of CRC cells (59). TAMs are the main cells in the tumor EMT (60). TAMs are related to the vascular system of CRC and can be used as markers of angiogenesis-mediated CRC. By studying 76 CRC patients, Marech et al. showed a significant correlation between macrophage infiltration and microvessel density (61). Furthermore, a large total number of TAMs is favorable for the CRC prognosis. Indeed, Nakayama et al. detected high levels of TAMs in patients with a good prognosis (62). Koelzer et al. found that CD68^+^ TAMs predicted longer OS (63). Similarly, Cavnar et al. reported a significant positive correlation between DFS and CD68^+^ cells in 188 patients with CRC liver metastasis (64). Compared with the total number of macrophages determined by CD68 markers, the M2-like phenotype of macrophages can better predict the adverse prognosis in CRC. In the study of Wei et al., high-level expression of interstitial CD163 at the front of tumor invasion was significantly correlated with tumor grade, lymphatic vascular invasion, tumor invasion, lymph node metastasis and TNM stage, and was associated with poor recurrence survival rate (RFS), as based on IHC analysis of 81 Chinese CRC patients (59). Yang et al. found that in 81 CRC patients, a high CD163^+^/CD68^+^ ratio at the front of tumor invasion (rather than at the tumor stroma) was closely related to enhance lymphatic vascular invasion, tumor invasion, TNM stage, RFS and OS in CRC patients (65).

### Myeloid-Derived Suppressor Cells (MDSCs)

Myeloid cells are composed of mononuclear myeloid cells and granulocytes, while mononuclear myeloid cells are mainly composed of monocytes, final differentiated macrophages and DCs. Granulocytes include neutrophils, eosinophils and basophils (66–68). In the early 1980s, these cells were found to be immunosuppressive. Therefore, to unify this group of cells, they were named bone marrow-derived suppressor cells in 2007 (69).

MDSCs interact with the TME, and tumor and stromal cells secrete TGF-β, MMP9, BV8, IL-6, IL-1β, β-FGF and VEFG through autocrine and paracrine mechanisms, mobilizing and expanding MDSCs and further promoting tumor growth (70). The TME can secrete chemokines, cause MDSCs to migrate to the tumor site, inhibit immune function and accelerate tumor progression (71, 72). CCL2 recruits MDSCs to the CRC TME (73), enhancing the immunosuppressive function by inhibiting T cell proliferation and stimulating Treg development (74). In a mouse model, reducing CXCL4 in CRC tumor tissue promoted the recruitment of MDSCs, resulting in an immunosuppressive environment and progression of CRC (75). Ouyang et al. found increased levels of CD33^+^ CD11b^+^HLA-DR^-^MDSCs in primary tumor tissues of CRC patients, which was related to advanced TNM stage and lymph node metastasis. At the same time, it was found that tumor cells induce the expansion of MDSCs through a variety of inflammatory factors. These tumor-derived MDSCs inhibit T cell proliferation and promote tumor cell growth through oxidative metabolism (76).

### Tumor-Associated Neutrophils (TANs)

Neutrophils are effector cells of the innate immune system. Unlike macrophages, neutrophils are not antigen-presenting cells but act as killer cells in the blood. Neutrophils are mainly produced in the bone marrow, accounting for 50-70% of human circulating leukocytes, with a half-life of only 5 days; however, they are the only immune cells that can dissolve cells and tissues (77). When the body releases chemokines after infection, neutrophils tend to migrate and recognize pathogens (78). In cancer, tumor cells and TAMs release the chemokines CXCL1/2/3/6/8 and CCL3/5, which induce neutrophils in peripheral blood to enter the TME and polarize into different TANs (79, 80).

A few studies on the relationship between TANs and the survival rate of CRC patients have been conducted (81). Rao et al. found that an increase in neutrophils in tumors is associated with a malignant phenotype and can predict poor prognosis in CRC (82). Galdiero et al. evaluated CRC patients receiving 5-FU chemotherapy and found that a higher TAN concentration was associated with better treatment efficacy. TANs are important immune cell infiltration components in CRC. In fact, evaluating TAN infiltration may help to identify patients who will benefit from 5-FU chemotherapy (83). Berry et al. analyzed the number of neutrophils in CRC tissues. Due to the lack of neutrophil-specific antibodies, neutrophils were counted manually according to their morphology, and high levels of TANs were associated with better overall survival (OS) in patients with stage II CRC (84). Furthermore, the combination of the neutrophil lymphocyte ratio and platelet count is able to predict the future clinical course of CRC (85). A high neutrophil to lymphocyte ratio (NLR) has also been shown to be a poor prognostic factor in CRC patients. Li et al. retrospectively analyzed a cohort of 354 patients with stage I-III CRC and observed a close relationship between dynamic changes in the NLR and the OS rate (86). Additionally, a high NLR has an adverse effect on the OS of CRC patients undergoing radical surgery (87).

### Tumor-Infiltrating Lymphocytes (TILs)

Lymphocytes are the main immune cells of tumors, including T, B, NK, and NKT cells, and these subsets can reflect tumor immunotherapy and serve as clinical biomarkers. T cells are the most abundant and characteristic immune cells in the TME and are divided into cytotoxic T cells, helper T cells, inhibitory T cells and NKT cells, in contrast to traditional T cells (88). T cells prevent tumor growth by targeting tumor cells. Tregs are a specific group of CD4^+^T cells related to the overreaction of immunosuppression, inflammation and allergic diseases (89). In cancers, Tregs are considered to inhibit immunity in most cases, and Treg infiltration is associated with poor prognosis (90–92). Marshall et al. found that Treg cells promote lung cancer metastasis (93). High FoxP3^+^ Treg infiltration exhibits a significant correlation with shorter OS patients with other solid tumors, including ovarian cancer, gastric cancer, renal cell carcinoma, melanoma, hepatocellular carcinoma, oral squamous cell carcinoma and breast cancer (94–97). However, in some tumors with chronic inflammatory infiltration, the accumulation of Tregs correlates positively with good prognosis. Frey et al. found that patients with mismatch repair deficiency (dMMR) CRC had high-level infiltration of Foxp3^+^ Tregs, with an increased survival rate (98). According to Hanke et al., high-level infiltration of Foxp3^+^ Tregs in early lymph node-negative CRC has a good prognosis (99). Vlad et al. also found that an increased Foxp3^+^ Treg density is associated with improved survival in CRC and is an independent prognostic factor (100). The relationship between Treg infiltration and tumor prognosis seems to be closely related to tumor type. Treg regulation plays the dual or multiple roles in antitumor immunity and the tumor treatment response, maintaining immune homeostasis and preventing autoimmunity (101, 102).

B lymphocytes participate in immune regulation mainly by producing immunoglobulin, presenting antigen secreting cytokines. B lymphocytes produce antibodies in the tumor microenvironment, which promotes tumor development (103, 104). B cells also inhibit tumor growth. Mouse B cells can promote antitumor activity through T cell-mediated immunity, inhibiting tumor development, and CD20-deficient mice show T cell antitumor inhibition (105–107). In malignant melanoma, enhanced patient survival is related to the simultaneous presence of tumor-related CD8^+^ T cells and CD20^+^ B cells but not to other clinical features (108). Research on the progression of CRC by B cells is limited, and views are inconsistent. There are differences between the B cell subsets in the peripheral blood, mesenteric lymph nodes and primary tumors of patients with CRC and those of healthy people, and B cells are activated in tumor-related tissues (109). After activation, B cells in patients with CRC differentiate into mature types, resulting in a specific response to tumors. On the other hand, the number of B cells in patients with metastatic CRC (mCRC) is small and the proportion of regulatory B cells is increased, which may be involved in immune escape (110, 111). Nevertheless, Berntsson et al. found that the survival time of CRC patients with B cell infiltration was prolonged (112). Through multiple-regions single-cell sequencing of tumors, normal mucosal tissue, liver metastases, and pairs of noncancerous tissues in CRC patients, a recent study showed that the contradictory effect of B cells on tumors in the past was due to the existence of multiple subtypes of B cells. IgA^+^IGCL2^+^ plasma cells are associated with poor prognosis of CRC, whereas highly proliferated GLC2^+^ plasma cells and circulating B cells are associated with a better prognosis (113).

Natural killer (NK) cells are effector cells of the immune system. When cells are infected or mutated by the virus, the expression of MHC-1 on their surface is lacking or abnormal. NK cells bind to NKG2D interaction ligands through antibody-dependent cell-mediated cytotoxicity (ADCC) or receptors, degranulate and release cytotoxic perforin and granzyme, induce signal transduction, and kill virus-infected cells and tumor cells (114). However, compared with those in adjacent normal tissues, NK cell levels in CRC tissues are low, suggesting that less NK cell infiltration may be one of the mechanisms of TME immune escape (115). The phenotype of peripheral NK cells in CRC patients changes, which promotes tumor progression (116). In CRC patients with curative tumor resection, the expression of NKp44 and NKG2D on circulating NK and NKT cells is increased, suggesting that the primary tumor and TME have an inhibitory effect on the phenotype of NK and NKT cells in CRC (117). *In vitro*, NK cells can enhance the cytotoxicity of cetuximab and the killing effect on RAS and BRAF mutant CRC cells (118). A phase I clinical trial observed NK cells to be closely related to the therapeutic effect of CRC. Cetuximab was significantly effective in patients with NK cell infiltration, though there was no significant correlation in patients who did not receive cetuximab (119). NK cell therapy has played a key role in hematological diseases and resulted in the use of NK cells in solid tumors. Initial results for chimeric antigen receptor-NK (CAR-NK) in the treatment of CRC patients have been obtained and NKG2D-CAR-modified NK cells showed antitumor effects in mouse models. At the same time, the standard CAR-NK was used in three CRC patients, who reached the safe end point (120). NK cells are also a prognostic factor for CRC recurrence (121).

In addition to NK cells and T cells, there is a special group of cells with the common characteristics of NK and T cells, called NKT cells. NKT cells have CD4^+^ CD8^+^ thymocytes, which develop in the thymus and migrate to peripheral organs such as the liver, spleen, lung and intestine (122, 123). Although NKT cells exert cytotoxicity, they mainly secrete a large number of helper T cytokines Th1-, Th2-, Th17-, Treg- or helper follicular cytokine (TFH)-cell related cytokines to play a regulatory role in innate or acquired immunity (124, 125). Type I NKT cells recognize glycosphingolipids α- galactose ceramide or its analogs (126, 127). α-Galactose ceramide (α-galcer) increases NKT expression and PD-1 in combination with α-galce increases the activity of NKT cells, enhances the antitumor response, and significantly reduces the occurrence of small and large intestinal tumors (128). Compared with the normal mucosa, the expression of CD69L and FasL is increased in infiltrating type I NKT cells in CRC tumor tissue, IFN- γ and granzyme B are also increased, and the OS rate of CRC patients with high-level NKT cell infiltration is higher than that of patients with low NKT cell infiltration (129). Intratumoral infiltration of NKT cells can be used as a prognostic factor for CRC.

### Exosomes

In the process of tumor cell growth, invasion and metastasis, tumor cells and interstitial fine cells located in the TME can not only secrete various soluble molecules, including cytokines and chemokines, but also release various vesicles. These vesicle structures are extracellular vesicle structures (130) that can be divided into exosomes (20-100 nm), microbubbles (100-1000 nm) and apoptotic bodies (1-5 µm) according to their size. Exosomes are different in size from microbubbles and apoptotic bodies and have specific surface molecular characteristics, such as CD9 and CD63 expression. Exosomes are present in almost all body fluids, including plasma/serum, saliva, breast milk, cerebrospinal fluid, urine and semen (131–141). Exosomes are also distributed in the TME and carry cargo including a variety of proteins, DNA, mRNA, miRNA, long noncoding RNA, and even virus/prion genetic material (142–146). Exosomes play a key role in local and remote intercellular communication in cancer and are an important part of the TME. Almost all kinds of cells in tumors can secrete exosomes, including tumor cells, tumor-associated adipocytes, TAFs, TAMs and vascular endothelial cells. Exosomes can be ingested by recipient cells to participate in intercellular signal exchange (130).

Zeng et al. found that in CRC, cancer-derived exosomal miR-25-3p promotes vascular permeability and angiogenesis by regulating the expression of VEGFR2, ZO-1, occludin and claudin-5 in endothelial cells by targeting KLF2 and KLF4. And miR-25-3p from CRC cells enhances CRC metastasis in the mouse liver and lungs. In addition, the expression of miR-25-3p in circulating exosomes is significantly higher in CRC patients with metastasis than in those without metastasis, and exosomes can be used as blood biomarkers (147). CRC-derived exosomal miR-106b-3p promotes tumor metastasis by downregulating DLC-1 expression (148). Exosomal miR-200c-3p negatively regulates the migration and invasion of CRC stimulated by lipopolysaccharide (LPS) (149). The CRC-derived exosomal circRNA, circPACR can be induced by miR-142-3p/miR-506-3p-TGF-β1 to promote CRC cell proliferation, migration and invasion (150). CAFs are the main components of the TME and promote cancer development through tumor matrix interactions. Bhome et al. compared the exosomes of normal and TAFs in CRC patients and found that CAFs were enriched in microRNAs 329, 181a, 199b, 382, 215 and 21, with microRNA 21 being the most abundant. After establishing the original transplanted tumor model with miR-21-overexpressing fibroblasts, liver metastasis increased (151). Exosomal miR-21 is expressed by stromal fibroblasts and promotes tumor cell metastasis. MiR-21 is involved in the progression of CRC. Exosomes secreted by CAFs are also involved in CRC metastasis and chemoresistance. Hu et al. found that CAFs secrete exosomes, resulting in a significant increase in the level of miR-92a-3p in CRC cells, activating the Wnt/β- catenin pathway and inhibiting mitochondrial apoptosis by directly inhibiting FBXW7 and MOAP1; the effect is to promote stemness, EMT, metastasis and 5-FU/L-OHP resistance in CRC (50). This finding provides an alternative way to predict and treat CRC metastasis and chemoresistance by inhibiting exosomal miR-92a-3p. Exosomes can also be used as diagnostic markers in CRC. Maminezhad et al. detected CRC cell lines and patient serum, and found increased levels of miR19a, miR-20a, miR-150 and let-7a but decreased levels of miR-143 and miR-145, with expression being related to TNM stage (152). Clinically, many miRNAs secreted by exosomes, such as let-7a, miR-1229, miR-1246, miR-150, miR-21, miR-223, and miR-23a have been used as diagnostic and prognostic markers for screening and predicting CRC tumors (153).

### Immunosenescence

Immunosenescence is a process of immune dysfunction that tend to cause inflammation and an immunosuppressive microenvironment leading to tumorigenesis and cancer metastasis (154). Senescence might become an obstacle to achieve efficacious immunotherapy in the TME, since senescent cells secret proinflammatory cytokines and growth factors, known as the senescence associated secretory phenotype (SASP), and this secretion has been implicated in both aging and cancer development (155). Giunco et al. found that in elderly CRC patients, senescent CD8 cells, but not CD4, displayed a significant relationship with disease outcome. Furthermore, the CD4/CD8 ratio was a prognostic marker of disease relapse in stage I-III CRC patients (156). In the TME of CRC, immunosenescent cells can influence the therapeutic effect since the majority of CRC patients with microsatellite stability (MSS) do not benefit from current anti-PD-1 therapy. A recent study found that in 18 MSS CRC patients, the number of immunosuppressive/exhausted T-cell phenotypes at tumor lesions were increased and CD8^+^ CD28^-^ immunosenescent T cells were accumulated according to single-cell mass cytometry analysis. Moreover, the TME of CRC hosts chemokines/cytokines that likely recruit immunosuppressive/exhausted T cell subsets to regulate the immune system (157). It is necessary to comprehensively understand the immunosenescence to help boost the immune response in patients with MSS CRC.

## Current Strategies Related to Immunotherapy in CRC

### Antiangiogenic Therapy

Bevacizumab, an immunoglobulin G monoclonal antibody against humanized vascular growth factor A, selectively binds to vascular endothelial factor subtype A (VEGF-A), hinders the binding of vascular endothelial growth factor to receptor tyrosine kinases (VEGFR), and initiates signaling to inhibit tumor angiogenesis (158, 159). Bevacizumab has been approved for first-line and second-line treatment of mCRC (160). An Italian randomized, open, multicenter, phase 3 clinical trial (NCT00719797) compared the efficacies of FOLFIRI combined with bevacizumab and FOLFIRI alone, and the median survival time of the FOLFIRI combined with bevacizumab group was greater than that of the FOLFIRI group (29.8 months *vs*. 25.8 months, HR = 0.80, *P* = 0.03) (161). Additionally, the combined use of bevacizumab did not significantly increase side effects but did enhance effective PFS and OS (162). The latest study found that bevacizumab combined with capecitabine was also effective as an advanced treatment for previously irinotecan-, oxaliplatin- and fluoropyrimidine-resistant mCRC (163). VEGF plays an important role in the CRC immune microenvironment, which can inhibit DC maturation, reduce T cell tumor infiltration and increase inhibitory cells in the TME (164–167). We found that the level of VEGF was increased in tumors. Moreover, bevacizumab inhibited the VEGF-VEGFR1 binding signal on DCs, NF-κB signaling, and DC cell maturation, prevented the increase in the amount of MHC and other molecules, and suppressed T cell activation. In CRC patients, bevacizumab elevated the number of mature DCs in the peripheral blood (168). Limagne et al. found that the amount pf MDSCs of patients decreased with FOLFOX in combination with bevacizumab, which was related to longer survival (169).

Ramucirumab, a humanized monoclonal antibody, mainly acts on the extracellular region of VEGF receptor 2 and has a beneficial role in gastric cancer, lung cancer and CRC (170–175). In the RAISE trial, after first-line oxaliplatin/fluoropyrimidine chemotherapy combined with bevacizumab for progressed CRC, ramucirumab was added to FOLFIRI, and the OS rate and progression-free survival(PFS) rate of patients were significantly improved (176).

Aflibercept is a monoclonal antibody composed of the extracellular segments of human VEGFR-1 and VEGFR-2 fused with the vascular endothelial growth factor-binding region and human immunoglobulin G1 FC region. Aflibercept β combined with FOLFIRI was approved for second-line treatment of mCRC in 2017 (177). A high-quality double-blind randomized controlled trial (RCT), the VELOUR trial, compared the efficacy of aflibercept plus FOLFIRI with that of placebo plus FOLFIRI, and the median OS, OS and PFS were higher than those in the former group (178). However, aflibercept in elderly patients (> 65 years old) shows a controllable increase in toxicity (179).

In addition to using monoclonal antibodies to inhibit the VEGFA pathway, some small molecule inhibitors have been applied in anti-VEGF therapy, such as regorafenib, sorafenib, sunitinib, pazopanib and axitinib. An international, multicenter, placebo-controlled phase III clinical trial (CORRECT) found that the median survival time of mCRC patients in the regorafenib group was longer than that in the placebo group (6.4 months *vs*. 5.0 months, HR = 0.77, *P* = 0.00052) (180). Regorafenib combined with nivolumab also has good applicability for the treatment of MSS chemotherapy-resistant mCRC (181). Sorafenib, a multi-kinase inhibitor that targets serine threonine and tyrosine kinases involved in tumor progression and angiogenesis, including all VEGFRs and PDGFR-β, RET, FLT3 and c-KIT (182), is used to treat advanced renal cell carcinoma, unresectable hepatocellular carcinoma and thyroid cancer (183–186). In CRC, a phase I clinical trial (RESPECT) found that the first-line combined use of sorafenib and oxaliplatin, folic acid and fluorouracil (mFOLFOX6) did not prolong PFS (187). In a multicenter, randomized phase II clinical trial (NEXIRI-2/PRODIGE 27), mCRC patients carrying RAS mutations had a prolonged 2-month no-progression rate and median PFS with the use of sorafenib combined with irinotecan after oxaliplatin, irinotecan, fluoropyrimidines and bevacizumab failed (188). Sunitinib is a small molecule multi-target receptor tyrosine kinase inhibitor. However, for patients with unresectable/advanced mCRC, the first-line combination of sunitinib and FOLFIRI did not lead to significant clinical benefits (189). A randomized, phase III clinical trial found no significant difference in median PFS between sunitinib combined with FOLFIRI and FOLFIRI combined with placebo (190). Fruquintinib, a small molecule selective VEGFR inhibitor independently developed in China, significantly prolonged the median OS of patients after three-line use of fruquintinib compared with that of patients receiving the placebo (9.3 months vs. 6.6 months, HR = 0.65) (191). The major antiangiogenic therapy agents under clinical investigation in CRC are summarized in **Table 1**.

**Table 1:** Summary of antiangiogenic therapy for colorectal cancer (CRC).

Regimen	Target population	Design	Treatment	Registration number	Reference
Bevacizumab	mCRC	Phase II	FOLFIRI+ Bevacizumab	NCT00467142	(192)
	mCRC	Phase II	Oxaliplatin + Bevacizumab + Capecitabine	NCT00159432	(193)
	mCRC	Phase II	FOLFOX + Bevacizumab FOLFOX + Bevacizumab + Irinotecan	NCT01321957	(194)
	mCRC	Phase II	Perioperative FOLFOXIRI + Bevacizumab Postoperative FOLFOX	NCT01540435	(195)
	mCRC	Phase III	FOLFOXIRI + Bevacizumab FOLFIRI + Bevacizumab	NCT00719797	(161)
	mCRC	Phase III	Capecitabine + Bevacizumab Capecitabine	NCT00484939	(196)
	mCRC	Phase III	Chemotherapy + Bevacizumab Chemotherapy + Cetuximab	NCT00265850	(197)
	mCRC	Phase III	Bevacizumab + Xelox	NCT00384176	(198)
	mCRC	Phase III	Bevacizumab + Chemotherapy Chemotherapy	NCT00112918	(199)
	mCRC	Phase III	Bevacizumab + Xelox	NCT00577031	(200)
Ramucirumab	mCRC	Phase III	Ramucirumab + FOLFIRI Placebo+ FOLFIRI	NCT01183780	(176)
Aflibercept	mCRC	Phase III	Aflibercept + FOLFIRI Placebo	NCT01661270	(201)
	mCRC	Phase III	Aflibercept + FOLFIRI Placebo	NCT00561470	(178, 202)
Regorafenib	mCRC	Phase III	Regorafenib Placebo	NCT01103323	(180)
	mCRC	Phase III	Regorafenib	NCT01853319	(203)
	mCRC	Phase III	Regorafenib Placebo	NCT01584830	(204)
Sorafenib	mCRC	Phase II	Sorafenib + Bevacizumab	NCT00826540	(182)
	mCRC	Phase II	Sorafenib + Irinotecan	NCT01715441	(188)
Sunitinib	mCRC	Phase II	Sunitinib + FOLFIRI	NCT00668863	(189)
	mCRC	Phase III	Sunitinib + FOLFIRI Placebo +FOLFIRI	NCT00457691	(190)
Fruquintinib	mCRC	Phase III	Fruquintinib Placebo	NCT02314819	(191, 205)

### Anti-EGFR Therapy

EGFR is a membrane-bound receptor tyrosine kinase protein that activates downstream intracellular signaling pathways, including MAPK (RAS/RAF/MEK/ERK), PI3K/AKT, and JAK/STAT3 signaling, and plays a role in tumor cell growth, proliferation and differentiation (206, 207). EGFR promotes tumor progression when overexpressed. CRC patients exhibit high-level expression of EGFR. Therefore, targeting EGFR and its downstream pathways has become a new strategy for the treatment of CRC (208). Cetuximab is a human/mouse chimeric IgG1 monoclonal antibody that mainly binds to EGFR on the surface of tumor cells and competitively blocks EGFR signaling to inhibit tumor cell proliferation. Cetuximab also inhibits the development of neovascularization by reducing the production of VEGF and activates the human anti- chimeric antibody (HACA) (209). Initial multiple clinical phase II trials found that among EGFR-positive patients, cetuximab combined with irinotecan had a better clinical effect than chemotherapy alone (210–212). Despite no significant difference between the PFS risk ratios and OS rates of mCRC patients receiving cetuximab combined with FOLFIRI and mCRC patients receiving FOLFIRI alone, cetuximab combined with FOLFIRI benefited KRAS wild-type patients (213). KRAS is an effector molecule responsible for signal transduction from ligand-bound EGFR to the nucleus. Activation of KRAS mutations often leads to CRC resistance to the EGFR targeted monoclonal antibodies (214). Therefore, EGFR-positive wild-type KRAS CRC responds to cetuximab (215). The CEBIFOX study found an ORR of 70.3%, a median PFS of 10.9 months (95% CI 9.0-12.9), and an OS of 33.8 months (95% CI 21.4-45.5) for fortnightly use of cetuximab combined with FOLFOX6 in patients with RAS wild-type mCRC (216).

Panitumumab, the first fully humanized IgG2 monoclonal antibody, displays a high affinity for EGFR, and its mechanism of action in CRC treatment is similar to that of cetuximab. Clinical phase II and III trials have shown that panitumumab can significantly prolong the PFS of patients with refractory CRC, with good tolerance (212, 217). In a randomized phase III trial (PRIME), the PFS of patients with wild-type KRAS was prolonged with mCRC first-line use of panitumumab combined with fluorouracil, folic acid and oxaliplatin (FOLFOX4) compared with that of patients receiving FOLFOX4 alone, though there was no significant difference in OS (218). In the randomized, open, phase II VOLFI study (AIO KRK0109), FOLFOXIRI combined with panitumumab was used as the first-line treatment for Ras wild-type mCRC improving the ORR and secondary surgical resection rate (219). In a phase II trial of locally advanced rectal cancer, FOLFOXIRI combined with panitumumab/cetuximab was used as a new adjuvant chemotherapy for patients with wild-type RAS-BRAF rectal cancer, with good clinical efficacy and tolerance (220). The major agents targeting EGFR therapy under clinical investigation in CRC are summarized in **Table 2**.

**Table 2:** Summary of anti-EGFR therapy for colorectal cancer (CRC).

Regimen	Target population	Design	Treatment	Registration number	Reference
Cetuximab	mCRC	Phase II	Cetuximab + FOLFOX-6	NCT01051167	(216)
	mCRC	Phase III	Chemotherapy + Bevacizumab Chemotherapy + Bevacizumab + Cetuximab	NCT00208546	(221)
	mCRC	Phase III	Cetuximab + FOLFIRI FOLFIRI	NCT00154102	(213)
Panitumumab	mCRC	Phase III	Panitumumab + FOLFOX4 FOLFOX4	NCT00364013	(218)
	mCRC	Phase II	Panitumumab + FOLFOXIFI FOLFOXIFI	NCT01328171	(219)

### Immune Checkpoint Inhibitors (ICIs)

Immune checkpoints are molecules that express and regulate the activation of immune cells. When the immune response is too strong, the immune checkpoint acts as a key regulator for attenuation (222). However, in cancer, immune checkpoints are highly activated and overexpressed; thus, antigens cannot be presented to T cells, inhibiting their immune function and resulting in malignant cell proliferation and tumor immune escape (223, 224). ICIs restore immune function mainly by targeting and/or blocking immune checkpoint protein ligands on the surface of T cells or other immune cell subsets (225). ICIs constitute a mature monoclonal antibody immunotherapy. The most widely studied immune checkpoint targets are programmed cell death 1 (PD-1) and cytotoxic T -lymphocyte-associated antigen 4 (CTLA-4), which are used for a variety of solid tumors (226–228). There are also studies on the potential role of other checkpoints in tumor immune regulation, such as lymphocyte activation gene-3 (LAG-3), T cell immunoglobulin-3 (Tim-3), T cell immunoglobulin and the ITIM domain (TIGIT) (229–233). CTLA-4 is a transmembrane protein that is mainly expressed on activated T cells and was first cloned in 1987 (234). CTLA-4 binds the B7 molecule to reduce T cell activity and inhibit T cell activation channels, with an immunosuppressive function (235, 236). In 2010, the CTLA-4 inhibitor ipilimumab was demonstrated to improve the long-term prognosis of patients with unresectable malignant melanoma (237). In 2011, ipilimumab became the first ICI approved by the FDA for cancer treatment. PD-1, a new member of the immunoglobulin gene superfamily, is expressed by various immune cells, such as CD4 and CD8 T cells, B cells, macrophages, DCs and tumor-infiltrating lymphocytes (TILs) (238, 239). PD-1 is a negative regulatory molecule that inhibits T cell activation and limits autoimmunity (240, 241). The use of PD-1/PD-L1 pathway inhibitors can restore the function of effector T cells, playing an antitumor role (242). At present, a variety of PD-1 and PD-L1 inhibitors have been approved by the FDA to treat a variety of tumors.

There are currently three PD-1 and CTLA-4 inhibitors approved by the FDA for CRC: pembrolizumab, nivolumab and ipilimumab (**Table 3**).

**Table 3:** FDA approved main agents of immune checkpoint inhibitors (ICIs) for colorectal cancer (CRC).

Regimen	Target population	FDA approved date	Registration number	Reference
PD-1 inhibitor				
Pembrolizumab	Unresectable or metastatic dMMR and high microsatellite instability (MSI-H) CRC	23 May 2017	NCT01876511 KEYNOTE-164 KEYNOTE-177 (NCT02563002)	(243–245)
Nivolumab	DMMR and MSI-H mCRC	1 August 2017	CheckMate 037 CheckMate 066 CheckMate 142 (NCT02060188)	(246–248)
PD-1 + CTLA-4 inhibitor				
Nivolumab + Ipilimumab	DMMR and MSI-H mCRC	10 July 2018	CheckMate 142	(249–251)

Pembrolizumab (Keytruda^®^) is the first PD-1 inhibitor approved by the FDA for metastatic malignant melanoma (252). In recent years, pembrolizumab has been used for non-small-cell lung cancer (253, 254), Hodgkin’s lymphoma (255, 256), HNSCC (257), urothelial carcinoma (258, 259), gastric cancer (260) and CRC (243, 244). The landmark clinical trial NCT01876511 for the treatment of CRC with pembrolizumab is noteworthy. The clinical trial included 11 dMMR CRC patients and 21 pMMR CRC patients and 9 patients with dMMR in other cancers. The immune-related objective response rate and immune-related PFS rate were 40% and 78% in dMMR CRC patients, and 0% and 11% in pMMR CRC patients, respectively. The median PFS and OS in the dMMR group were not achieved, and the median PFS and OS in the pMMR CRC group were 2.2 months and 5.0 months, respectively (HR = 0.1, *P* < 0.001) (245). Based on these data, pembrolizumab (Keytruda^®^) was approved to treat unresectable or metastatic dMMR and high microsatellite instability (MSI-H) CRC by the FDA on May 23, 2017 (261). KEYNOTE-164 is a phase II clinical trial for evaluating pembrolizumab in the treatment of refractory, MSI-H/dMMR metastatic CRC. At the end of the trial data, the median follow-up time of group A (previously received ≥ 2-line treatment) was 31.3 months (range of 0.2-35.6 months), the objective response rate was 33% (95% CI, 21% - 46%), and the median PFS was 2.3 months (95% CI, 2.1-8.1 months). The median follow-up time of CRC in group B (previous ≥ 1-line treatment) was 24.2 months (range of 0.1-27.1 months), the objective response rate was 33% (95% CI, 22% - 46%) and the median PFS was 4.1 months (95% CI, 2.1-18.9 months). The incidence of treatment-related grade 3-4 adverse events was 16% in group A and 13% in group B. Thus, pembrolizumab can be safely used in patients with MSI-H/dMMR CRC (244). KEYNOTE-177 (NCT02563002) is a phase III clinical trial in which patients with metastatic MSI-H/dMMR CRC were randomly assigned to the pembrolizumab arm, though patients receiving chemotherapy could switch to pembrolizumab if disease progression occurred. The PFSs of the pembrolizumab and chemotherapy groups were 16.5 months and 8.2 months, respectively (HR = 0.60; 95% CI, 0.45-0.80; *P* = 0.0002) (243).

Another successful PD-1 inhibitor is nivolumab (Opdivo^®^). Based on the CheckMate 037 and CheckMate 066 trials, nivolumab has also been approved for the first time to treat unresectable or metastatic melanoma (246, 247). Nivolumab showed a good therapeutic effect in mCRC patients with dMMR/MSI-H. CheckMate 142 (NCT02060188) found that 23/74 patients achieved objective remission, and 68.9% (51/74) of patients received > 12 weeks of disease control; the safety of dMMR/MSI-H mCRC was tolerable (248). Nivolumab was approved on August 1, 2017, for dMMR and MSI-H mCRC. Interestingly, the CTLA inhibitor ipilimumab has also shows a certain therapeutic effect in CRC. Ipilimumab combined with nivolumab as the treatment for dMMR/MSI-H mCRC patients was effective at 9 months in 94% of patients; the PFS rates at 12 months were 76% and 71% for dMMR and MSI-H mCRC patients, respectively, and the OS rates at 12 months were 87% and 85% for dMMR and MSI-H mCRC patients, respectively (249). Moreover, ipilimumab combined with nivolumab did not significantly increase toxicity or side effects (250). Therefore, ipilimumab and nivolumab were approved for dMMR and MSI-H mCRC on July 10, 2018. Furthermore, a recent phase II CheckMate 142 study found that first-line nivolumab plus low-dose ipilimumab had robust and durable clinical benefit and was well tolerated as a first-line treatment for MSI-H/dMMR mCRC patients (251).

### Adoptive Cell Therapy (ACT)

Adoptive cell therapy utilizes the immune cells, such as T cells, DCs, NK cells or cytokine-induced killer (CIK) cells, of patients or other donors for tumor patients to achieve anti-tumor effects. ACT for CRC treatment includes TILs, CIK cell therapy and chimeric antigen receptor-modified (CAR) T cell therapy (**Table 4**). In a clinical study on TILs combined with IL-2 in CRC, patients in the ACT group received TILs extracted from metastatic tumors, as stimulated and amplified with high-dose IL-2, whereas the control group received traditional chemotherapy. Although no significant difference in disease-free survival (DFS) was observed between the two groups after 1, 3 and 5 years, TCRϵ chain expression increased significantly in disease-free patients compared with that in patients with recurrence (*P* = 0.04), suggesting that TILs play a role in the immune response (262). Sentinel lymph node (SLN)-T cells are also used for ACT in the treatment of CRC. A preliminary study found that after 16 CRC patients were injected with SLN T cells, 4 of 9 patients with stage IV CRC experienced complete remission, with a median survival time of 2.6 years, which was much greater than the median survival time of 0.8 years in the control group (263). In another I/II clinical study of 55 patients with CRC with SLN metastasis, the median OS of the experimental group that received expanded lymphocytes was 28 months, whereas that of the control group was 14 months, and no obvious toxicity or side effects was observed (264).

**Table 4:** Summary of adoptive cell therapy (ACT) for colorectal cancer (CRC).

Regimen	Target population	Design	Registration number	Status
TILs	Malignant Solid Neoplasm including MCRC Adenocarcinoma, Metastatic Ovarian Carcinoma	Phase II	NCT03610490	Active, not recruiting
	CRC stage III	Phase I/II	NCT03904537	Recruiting
	CRC	/	NCT02980146	Completed
CEA-CAR-T	CRC stage III, CRC liver metastasis	Phase I	NCT04513431	Not yet recruiting
	Solid cancers including CRC	Phase I, II	NCT04348643	Recruiting
	CRC Peritoneal Carcinomatosis Peritoneal Metastases Gastric Cancer Breast Cancer Pancreas Cancer	Phase I	NCT03682744	Active, not recruiting
CART72	mCRC	Phase I	CC-9701 and CC-9702 clinical trials by Cell Genesys and Aventis Pharmaceuticals	Completed
CIK	CRC	Phase IV	NCT03084809	Completed
DC-CIK	CRC	Phase II, III	NCT02419677	Completed
	CRC	Phase II, III	NCT02415699	Unknown
	CRC	Phase II	NCT02202928	Unknown

Many clinical trials using TILs or SLN T cells as a treatment for CRC (NCT03610490, NCT03904537, and NCT02980146) are being performed. NK cells have natural cytotoxicity toward tumor cells, with antibody dependent cytotoxicity (ADCC), and can secrete a variety of cytokines and chemokines for an immunomodulatory role. Therefore, NK cells can also be used for adoptive therapy. In a phase I clinical trial, expanded NK cells combined with IgG1 antibody were used to treat patients with gastric cancer or CRC. Among 6 evaluable patients, 4 were in stable condition (SD), and disease progression occurred in two patients (119). As TILs have the limitation of needing to be expanded from a tumor, exogenous T cell receptors (TCRs) have been expressed on cells by genetic engineering technology. Carcinoembryonic antigen (CEA) levels are often elevated in the tissues and serum of patients with gastrointestinal tumors. Therefore, genetically engineered autologous T lymphocytes that express mouse TCR against human CEA have been used for CRC treatment. In a phase I clinical trial, three patients with refractory mCRC were administered TCR targeting CEA, and their serum CEA levels were significantly decreased (74–99%). One patient showed reduced liver and lung metastases, but all three patients experienced severe transient inflammatory colitis (265). CAR-T cells have achieved remarkable results in B-cell leukemia and lymphoma, although the development of solid tumors is lagging (266, 267). A phase I clinical trial of targeted CEA-CAR-T cells for CRC treatment found that 7 of the 10 CEA^+^ patients were in stable condition after CAR-T cell treatment, with 2 patients maintaining this state for more than 30 weeks, and 2 patients underwent tumor regression (268). Another study on CAR-T cells targeting tumor-associated glycoprotein (TAG)-72 (CART72 cells) in the treatment of mCRC found that a very short duration of CART72 cells in the blood (≤14 weeks), suggesting that CART72 cells have a limited role in mCRC (269). A CAR-NK study targeting NKG2D found that after three mCRC patients received local infusion of CAR-NK cells, ascites production decreased and tumor cells in ascites samples decreased significantly. In addition, the method using RNA to make CAR can enhance the specificity of NK cells to NKG2DL and their tumor cell killing activity (120).

CIK cells treatment is a part of ACT and is induced by mononuclear cells cultured with CD3 monoclonal antibody and cytokines such as IFN-γ, IL-1 and IL-2. CIK cells include activated NKT cells, CD3^-^/CD56^+^ NK cells and CD3^+^/CD56^-^ CTLs. CIK cells have the characteristics of rapid proliferation, strong antitumor activity, wide spectrum and low toxicity. They have been used in the treatment of various solid tumors, such as hepatocellular carcinoma, renal cell carcinoma, gastric cancer, breast cancer, ovarian cancer, non-small-cell lung cancer and nasopharyngeal carcinoma. The efficacy of somatic CIK cells in patients with mCRC was examined in a phase II clinical trial. MCRC patients in the experimental group received chemotherapy combined with CIK cells, whereas the control group received chemotherapy alone. The median OS rates of the experimental and the control groups were 36 months and 16 months, respectively (*P* < 0.001), and the PFS rates were 16 months and10 months (*P* = 0.072), respectively. Although there was no significant difference, there was an increasing trend (270). A retrospective study using CIK cells to treat postoperative CRC patients reported a median PFS and median OS in the CIK group of 25.8 months and 41.3 months, respectively, while the median PFS and median OS in the control group were 12.0 months and 30.8 months, respectively (271). Another retrospective study on the efficacy of postoperative adjuvant infusion of CIK cells combined with chemotherapy for CRC observed a significantly longer DFS in the group than in the control group [HR = 0.28, 95% CI (0.09, 0.91), *P* = 0.034]. The 2-year DFS rates of the CIK group and control group were 59.65 ± 24.80% and 29.35 ± 6.39%, respectively. Moreover, CIK cell infusion was not associated with immediate adverse reactions (272). Dendritic cytokine-induced killer cells (DC-CIK) were observed in the combined first-line treatment of advanced CRC. The 5-year OS rates of the DC-CIK group and non-DC-CIK group were 41.3% and 19.4% (*P* = 0.001), and the 5-year PFS rates of the DC-CIK group and non-DC-CIK group were 57.4% and 33.6% (*P* = 0.022), respectively (273). Overall, DC-CIK immunotherapy combined with first-line treatment can significantly prolong the 5-year OS and PFS rates in patients with advanced CRC.

### Cancer Vaccine and Oncolytic Virus Therapy

Cancer vaccines are another method of immunotherapy for CRC (**Table 5**). Tumor cells express tumor-associated antigen (TAA), and by expressing specific tumor antigens, cancer vaccines can stimulate the body to produce a specific immune response. However, the results obtained for vaccines in the treatment of CRC are not consistent. Initially, a prospective randomized controlled clinical trial was conducted in CRC patients, and an autologous tumor cell BCG vaccine that induced active specific immunotherapy (ASI) was used. The study found that there was no statistically significant difference in the survival rate or disease-free survival rate of 80 eligible patients (279). A randomized phase III clinical trial of adjuvant ASI with autologous tumor cell BCG reported no significant difference in DFS and OS rates between the surgical resection plus ASI group and the simple resection group of stage II and III CRC patients (280). In another study of stage II and III CRC patients, despite no clinical benefit of autologous tumor cell BCG immunotherapy for stage III CRC after surgery, the recurrence-free period of autologous tumor cell BCG adjuvant ASI after surgical resection was significantly longer than that of simple resection (*P* = 0.011), and recurrence-free survival was significantly prolonged (*P* = 0.032) (281). As antigen-presenting cells, DCs are also often modified to produce vaccines. A randomized trial for CRC patients on the activation of CD40L by DC vaccines *in vitro* found that 15 of 24 patients had immune induction reactions. The five-year recurrence-free survival rate (RFS) of those who had tumor-specific T cell proliferation or IFN-γ induced by the vaccine that appeared at one week after vaccination was significantly higher than that of patients without a response (63% *vs*. 18%, *P* = 0.037) (282). The randomized phase II clinical trial on administering DC vaccines after complete resection of CRC liver metastasis showed a significantly longer median DFS for the vaccine group was than for the observation group (25.6 months *vs*. 9.53 months) (283).

**Table 5:** Summary of cancer vaccine and oncolytic virus therapies for colorectal cancer (CRC).

Regimen	Target population	Design	Registration number	Reference
AD5 CEA Vaccine	CRC Lung Cancer Breast Cancer	Phase I, II	NCT01147965	(274)
CEA (6D) VRP Vaccine (AVX701)	CRC Lung Cancer Breast Cancer Pancreatic Cancer Colon Cancer	Phase I, II	NCT00529984	Completed
	CRC stage III	Phase I	NCT01890213	Completed
DC/PANVAC or GM-CSF	CRC Metastasis Cancers	Phase II	NCT00103142	(275)
Modified vaccine Ankara–5T	mCRC	/	ISRCTN54669986	(276)
GVAX Colon Vaccine	mCRC	Phase II	NCT02981524	Completed
MVA-Brachyury-TRICOM	CRC	Phase I	NCT02179515	(277)
Bavarian Nordic (BN)-CV301	CRC	Phase I	/	(278)

Guanylcyclase C (GUCY2C), which is selectively expressed by intestinal epithelial cells and some neurons, is almost universally overexpressed in CRC (284). According to a phase I study using the Ad5-GUCY2C-PADRE vaccine in the treatment of stage I or II (pN0) colon cancer (NCT01972737), the vaccine can stimulate the immune response of T cells and has certain safety (285). CEA is overexpressed in CRC and acts as a tumor antigen marker. A phase I/II trial using the Ad5[E1-, e2b-]-CEA (6D) vaccine for advanced CRC reported a median survival time for the 32 patients included in the study of 11 months, and the Ad5 [E1-, e2b -] - CEA (6D) vaccine was well tolerated and induced an immune response (286). In the phase I study of patients with stage III CRC treated with virus-like replicator particle (VRP)-CEA, 12 CRC patients completed standard postoperative adjuvant chemotherapy and received VRP-CEA immunization 4 times every 3 weeks. The 5-year RFS rate was 75% (95% CI 40-91%), and no deaths were observed during the period. After vaccination, levels of CD8^+^ TEMs increased (10/12), Foxp3^+^ Tregs decreased (10/12), and specific CEA and IFN-γ produced by CD8^+^ granzyme B^+^ TCM cells increased (287).

Several CRC clinical trials of CEA-modified tumor vaccines have been carried out, such as NCT01147965, NCT00529984, and NCT01890213 (274). MUC1 is abnormally expressed in tumors and is also a tumor-associated antigen. DCs and poxvirus vectors act as immune stimulants against tumor antigens. A randomized phase II trial (NCT00103142) compared whether two vaccines based on DCs and pox vectors encoding CEA and MUC1 (PANVAC) can prolong the survival of mCRC patients after resection (275). Seventy-four mCRC patients after resection and perioperative chemotherapy were randomly treated with autologous modified PANVAC with DC (DC/PANVAC) or GM-CSF (granulocyte macrophage colony-stimulating factor) every time. The 2-year RFS rates of the two groups were similar, and the DC and poxvirus vectors had similar activity. As a treatment for mCRC, the modified vaccine Ankara-5T4 and low-dose cyclophosphamide improved the antitumor immune response and prolonged survival, with no safety problems (276). Furthermore, a phase I trial (NCT02179515) was performed to test the safety and tolerability of a modified vaccinia Ankara (MVA)-based vaccine modified to express brachyury and T-cell costimulatory molecules (MVA-Brachyury-TRICOM) in advanced patients including colon cancer patients. Heery et al. found that the MVA-brachyury-TRICOM vaccine directed against a transcription factor known to mediate EMT can be administered safely in patients with advanced cancer and can activate brachyury-specific T cells *in vitro* and in patients (277). Recently, a phase I dose-escalation trial of Bavarian Nordic (BN)-CV301, which is a recombinant poxviral vaccine targeting MUC-1 and CEA with costimulatory molecules, was conducted to test the safety and immune response of the vaccine. The trial found that the BN-CV301 vaccine was safely administered to patients with advanced cancer (278).

GVAX is a cellular immunotherapy induced by an allogeneic, whole-cell, granulocyte macrophage colony-stimulating factor that can induce the immune response of T cells to TAAs. A phase 2 study (NCT02981524) of the colon GVAX vaccine, cyclophosphamide and pembrolizumab in 17 patients with pMMR advanced CRC was carried out. The median PFS was 82 days (95% CI 48-97 days), and the median OS was 213 days (95% CI 179-441 days) (288). Although GVAX/Cy plus PD-1 did not achieve the main outcome expected in pMMR CRC, biochemical reactions were observed in patients, providing a certain method to cause insensitivity to PD-1 in pMMR CRC, which still needs to be further explored in combination with other drugs. Although there is no cancer vaccine approved for clinical use, a large number of clinical trials are ongoing and are expected to further improve the therapeutic effect on CRC.

### Tumor-Derived Exosomes Therapy

Tumor-derived exosomes have a certain potential antigenicity and can induce a strong antitumor immune response (289). Therefore, in addition to being a potential diagnostic marker, some studies have found that these exosomes can play a role as vaccines in CRC. A phase I clinical trial included 40 patients with HLA-A0201^+^ CEA^+^ advanced CRC who were randomly treated with AEX (ascites-derived exosomes) or AEX plus GM-CSF, and both methods were safe and tolerable. The patients in the AEX plus GM-CSF group showed a strong tumor-specific anti-tumor cytotoxic T lymphocyte reaction. These data suggest that immunotherapy with AEX plus GM-CSF can be used as an effective vaccine for mCRC patients (290).

## Conclusion and Prospects

The TME is a complicated landscape that is not only closely related to the growth and development of CRC but also affects the treatment and prognosis of patients with colorectal cancer. A variety of cytokines, chemokines, matrix enzymes and immunosuppressive cells, such as Tregs and MDSCs, shape the immunosuppressive environment of CRC. Although immunotherapy has achieved good results in malignant melanoma and lung cancer, its results in CRC are still poor. Therefore, it is particularly important to deeply study the TME, reverse or prevent tumor immune resistance and find a better way to treat CRC. At present, research on anti-PD-1 antibodies, adoptive cell immunotherapy, vaccine therapeutics and oncolytic viruses is being carried out. We need to carry out more clinical experiments, find more biomarkers for CRC, and make rational use of the differences in immune typing and genotyping of CRC such that suitable patients can benefit from immunotherapy.

## Author Contributions

The manuscript was conceptualized by YC and CW. YC wrote the majority of the manuscript and produced the tables. XZ helped to write the manuscript. CW critically revised the manuscript. All authors contributed to the article and approved the submitted version.

## Funding

This work was supported by grants from the National Natural Science Foundation of China (No. 31570908).

## Conflict of Interest

The authors declare that the research was conducted in the absence of any commercial or financial relationships that could be construed as a potential conflict of interest.

## Publisher’s Note

All claims expressed in this article are solely those of the authors and do not necessarily represent those of their affiliated organizations, or those of the publisher, the editors and the reviewers. Any product that may be evaluated in this article, or claim that may be made by its manufacturer, is not guaranteed or endorsed by the publisher.

## Abbreviations

CRC, colorectal cancer; ICIs, immune checkpoint inhibitors; TME, tumor immune microenvironment; DCs, dendritic cells; ECM, extracellular matrix; VEGF, vascular endothelial growth factor; VEFGR, vascular endothelial growth factor receptor; TGF-β, transformation and growth factors-β; PDGF, platelet-derived growth factor, CAF, cancer associated fibroblasts; TAM, tumor-associated macrophage; TAN, tumor-associated neutrophil; MDSC, myeloid-derived suppressor cell; TILs, tumor-infiltrating lymphocytes; mCRC, metastatic CRC; NK, Natural killer; OS, overall survival; PD-1, programmed cell death 1; CTLA-4, cytotoxic T-lymphocyte-associated antigen 4; ACT, adoptive cell therapy; MSS, microsatellite stable; dMMR, mismatch repair deficieny; MSI-H, high microsatellite instability; CAR, chimeric antigen receptor-modified; CIK, cytokine-induced killer; OS, overall survival; PFS, progression-free survival; DFS, disease-free survival; RFS, recurrence-free survival rate.
